# Supplementary material for: Cost-effectiveness of the ONCORAL multidisciplinary programme for the management of outpatients taking oral anticancer agents at risk of drug-related event: protocol for a pragmatic randomised controlled study
Source: BMJ Open. 2024 Feb 17;14(2):e074956. doi: 10.1136/bmjopen-2023-074956 (PMC10875583; doi:10.1136/bmjopen-2023-074956)
Supplement: Supplementary data [file bmjopen-2023-074956supp001.pdf]

Supplementary table: Overview of the course of the study

STUDY PERIOD	*Enrolment & Allocation*	*Allocation*													*Close out*
TIMEPOINT	D_0	D_8 † +/- 3d	D_{15} +/- 3d	D_{22} +/- 3d	M_1 +/- 8d	M_{1+15j} † +/- 3d	M_2 +/- 8d	M_{2+15d} † +/- 3d	M_3 +/- 8d	M_4†, M_5† +/- 8d	M_6 +/- 15d	M_7, M_8 +/- 15d	M_9 +/- 15d	M_{10}, M_{11} +/- 15d	M_{12} +/- 15d
ON-SITE VISIT	*V1*				V_2		V_3		V_4		V_5		V_6		V_7
**ENROLMENT:**															
Informed consent	X														
Randomisation	X														
Background and socio-demographic information	X														
Patient booklet (delivery, then collection of the data)	X				X		X		X		X		X		X
**INTERVENTION:**<br>**ONCORAL educational session***	X	X			X	X	X	X	X	X	X		X		X
Information on treatment by the community pharmacist in conjunction with the hospital pharmacist*	X				X		X		X		X		X		X
Information on treatment by the community pharmacist*										X		X		X	
Micro-costing*	X	X	X	X	X	X	X	X	X	X	X	X	X	X	X
**ASSESSMENTS:**															
Resource consumption					X		X		X		X		X		X
Prescription changes for the oral anticancer drug, if any					X		X		X		X		X		X
Adherence scale									X		X				X
Prescription refill rate									X		X				X
Adverse events		X*	X*	X*	X	X*	X	X*	X		X		X		X
Medication errors					X		X		X		X		X		X
Drug-drug interactions					X*		X*		X*		X*		X*		X
EQ-5D-3L	X				X		X		X		X		X		X
EORTC QLQ-C30	X								X		X				X
SatMed Q					X				X		X		X		X
Brief-IPQ	X										X				X

BMQ	X										X				X
Perceived social support	X										X				X
Health locus of control questionnaire	X										X				X
Vital status					X		X		X		X		X		X
Patient and health professional satisfaction with the ONCORAL programme*															X

* Only for patients randomised in the intervention group (ONCORAL programme)
† Patients in the intervention group are reached by phone for this session
BMQ = Beliefs about Medicines Questionnaire. Brief IPQ = Brief Illness Perception Questionnaire. EQ-5D-3L = EuroQool-5Dimension questionnaire. OAA = Oral anticancer agents. SATMED-Q = Treatment Satisfaction with Medicines questionnaire. V = Visit.
