# Supplementary material for: Cost-effectiveness of the ONCORAL multidisciplinary programme for the management of outpatients taking oral anticancer agents at risk of drug-related event: protocol for a pragmatic randomised controlled study
Source: BMJ Open. 2024 Feb 17;14(2):e074956. doi: 10.1136/bmjopen-2023-074956 (PMC10875583; doi:10.1136/bmjopen-2023-074956)
Supplement: Author's manuscript [file bmjopen-2023-074956.draft_revisions.pdf]

BMJ Open

BMJ Open is committed to open peer review. As part of this commitment we make the peer review history of every article we publish publicly available.

When an article is published we post the peer reviewers' comments and the authors' responses online. We also post the versions of the paper that were used during peer review. These are the versions that the peer review comments apply to.

The versions of the paper that follow are the versions that were submitted during the peer review process. They are not the versions of record or the final published versions. They should not be cited or distributed as the published version of this manuscript.

BMJ Open is an open access journal and the full, final, typeset and author-corrected version of record of the manuscript is available on our site with no access controls, subscription charges or pay-per-view fees (http://bmjopen.bmj.com).

If you have any questions on BMJ Open's open peer review process please email info.bmjopen@bmj.com

BMJ Open

Cost-effectiveness of the ONCORAL multidisciplinary programme for the management of outpatients taking oral anticancer agents at risk of drug related event: protocol for a pragmatic randomised controlled study

Journal:	*BMJ Open*
Manuscript ID	bmjopen-2023-074956
Article Type:	Protocol
Date Submitted by the Author:	24-Apr-2023
Complete List of Authors:	Huot, Laure; Hospices Civils de Lyon, Pole Sante Publique; Université Lyon 1, U1290 INSERM RESHAPE Research on Healthcare Performance<br>Guerre, Pascale; Hospices Civils de Lyon, Pôle Santé Publique, Service Evaluation Economique en Santé; Université Lyon 1, UR4129 Parcours Systémique en Santé P2S<br>DESCOTES, Guillaume; Hospices Civils de Lyon, Unité de Pharmacie Clinique Oncologique<br>CAFFIN, Anne-Gaëlle; Hospices Civils de Lyon, Unité de Pharmacie Clinique Oncologique<br>HERLEDAN, Chloé; Hospices Civils de Lyon, Unité de Pharmacie Clinique Oncologique; Université Lyon 1, EA3738 CICLY Centre pour l'innovation et la cancérologie de Lyon1<br>Ranchon, Florence; Hospices Civils de Lyon, Groupement Hospitalier Sud, Clinical Oncology Pharmacy Department; Université Lyon 1, EA3738 CICLY Centre pour l'innovation et la cancérologie de Lyon1<br>Rioufol, Catherine; Hospices Civils de Lyon, Groupement Hospitalier Sud, Clinical Oncology Pharmacy Department; Université Lyon 1, EMR 3738
Keywords:	Adult oncology < ONCOLOGY, Health economics < HEALTH SERVICES ADMINISTRATION & MANAGEMENT, Quality of Life

SCHOLARONE™
Manuscripts

TITLE: Cost-effectiveness of the ONCORAL multidisciplinary programme for the management of outpatients taking oral anticancer agents at risk of drug related event: protocol for a pragmatic randomised controlled study

AUTHORS: Laure HUOT[1,2], Pascale GUERRE[1,3], Guillaume DESCOTES[4], Anne-Gaëlle CAFFIN[4], Chloé HERLEDAN[4.5], Florence RANCHON[4.5,†], Catherine RIOUFOL[4.5,†]

1. Hospices Civils de Lyon, Pôle de Santé Publique, Service Evaluation Economique en Santé, F-69003 Lyon, France

2. Université Lyon 1, Inserm U1290 Research on Healthcare Performance (RESHAPE), F-69008 Lyon, France

3. Université Lyon 1, Laboratoire Parcours Systémique en Santé, F-69003 Lyon, France

4. Hospices Civils de Lyon, Unité de Pharmacie Clinique Oncologique, F-69495 Pierre-Bénite, France

5. Université Lyon 1, CICLY Centre pour l'Innovation et la Cancérologie de Lyon 1-EA3738, F-69921 Lyon, France

† Both authors contributed equally

Corresponding author:

Pr. Catherine RIOUFOL

Hospices Civils de Lyon, Hôpital Lyon Sud, Service pharmaceutique, Unité de Pharmacie Clinique Oncologique, 165 Chemin du Grand Revoyet, F-69495 PIERRE-BENITE, FRANCE

catherine.rioufol@chu-lyon.fr

KEYWORDS

Oral anticancer agent (OAA); Medication management programme; Study protocol; Cost-effectiveness

WORD COUNT: 3 381

For peer review only

ABSTRACT

Introduction

The development of oral anticancer agents (OAA) has profoundly changed cancer care, leading patients to manage their chemotherapy treatment on an outpatient basis. The prevention of iatrogenic effects of OAA remains a major concern, especially since their side effects are not less serious than those of intravenous chemotherapy. The ONCORAL programme was set up to secure the management of OAA in cancer patients followed at the Lyon University Hospital. This multidisciplinary programme involves hospital pharmacists, nurses, oncologists, and haematologists, as well as community health professionals. Given the economic stakes that this programme entails for the health system, a medico-economic study was designed.

Methods and analysis

This is a prospective controlled study, with individual open-label randomisation. A total of 216 outpatients treated with OAA and at risk of developing a drug-related iatrogenic event, will be randomised (2:1) to undergo follow-up in the ONCORAL programme or usual care. The primary outcome will be the estimation of the incremental cost-effectiveness ratio (difference in total costs per quality adjusted life years gained) at 12 months between the 2 groups. The secondary outcomes will be evaluation of OAA management consequences (relative-dose intensity, adherence, adverse drug events, drug-drug interactions, and proven medication errors), evaluation of overall survival and cancer-related quality of life, and patient reported outcomes in relation to the treatment. A budget impact analysis will be implemented. Patient and health professional satisfaction regarding the ONCORAL programme will be measured.

Ethics and dissemination

Approval to conduct this study was obtained from an Ethics Committee (*Comité de Protection des Personnes Ile-de-France VI*) in October 2019, and from the French data protection agency (*Commission Nationale de l'Informatique et des Libertés*), according to the

For peer review only

French Law. Trial results will be disseminated at clinical conferences and published in peer-reviewed journals.

Trial registration: NCT03660670; recruitment started in December 2020.

For peer review only

STRENGTHS AND LIMITATIONS OF THIS STUDY

- This study is the first cost-effectiveness evaluation of a multidisciplinary medication management programme for adult cancer outpatients treated with OAA.
- A pragmatic open-label randomised design was adopted, based on a programme implemented in a French University Hospital, involving hospital health professionals (oncologists, haematologists, pharmacists, and nurses) and based on a structured relationship with the community health professionals who follow the patient.
- The cost-effectiveness will be estimated at 12 months, by comparing the difference in total costs and quality of life-adjusted years (QALYs) gained between the ONCORAL programme and usual care.
- Due to the nature of the interventions, patients and professionals participating in the study are not blinded to management allocation.

For peer review only

INTRODUCTION

For many years, the treatment of cancer patients remained essentially hospital-based, relying on surgery, radiotherapy, and injectable chemotherapy. Since the early 2000's, oral anticancer agents (OAA), mostly targeted therapies such as tyrosine kinase inhibitors, have been steadily developed, contributing to significantly improve the prognosis of cancer patients.[1]

By allowing patients previously treated by injectable chemotherapy to receive oral therapy, the use of OAA has shifted patient management from hospital and healthcare provider administered treatment to self-administered treatment at home.[2] Despite their high price, OAA were expected to reduce certain healthcare costs and were considered for their convenience and ease of administration.[3 4]

However, these drugs expose patients to iatrogenic risks, in part due to their narrow therapeutic index and complex pharmacological profile.[5] They may also result in drug-related problems such as medication errors, adverse effects, and drug-drug interactions, which have been reported in half of the patients and considered as major in almost 20%.[6] Importantly, OAA adherence may be reduced due to these drug-related problems and may be further complicated by the administration regimen, such as a discontinuous dosing regimen or combination with another OAA or injectable chemotherapy.[7 8] In this context, the prevention of iatrogenic effects is crucial to enhance safety. Furthermore, as drug-related problems lead to an over-consumption of healthcare resources, this could lead to an increase in patient management-related costs.

It has been reported that patients show a preference for OAA over injectable chemotherapy, as the former meets their expectations in terms of comfort and autonomy.[9 10] Their perception of efficacy and past treatments experience (including toxicity) may also influence this preference.[2] It has been shown that for patients to successfully manage their OAA treatment, self-care support and multidisciplinary education across healthcare settings are needed.[11]

For peer review only

Finally, such medication management contributes to the optimal effectiveness of the treatment and a better quality of life.[12 13]

Structured OAA programmes have been set up in different countries and described in the literature, based on different components and organisation,[14] highlighting the follow-up and monitoring of patients throughout their treatment.[15] A promising effect has been described in the literature for certain adherence enhancing interventions, applied to target one or multiple factors influencing patient behaviour.[16] The management of side effects should be more specifically included in OAA care programmes in order to help patients better identify them and improve their self-care behaviour.[11] Although studies have also assessed the economic impact of pharmacist-led medication management programmes, showing cost savings and cost avoidance,[17 18] none has evaluated the efficiency of such interventions on the whole patient pathway. There is thus still a need for rigorous studies to assess educational and counselling practices supporting adherence to OAA in terms of clinical benefit and efficiency.[19]

We set up a medico-economic study to evaluate, with a high level of evidence, the cost-effectiveness of a multidisciplinary OAA management programme for outpatients with cancer followed in a French university hospital.

METHODS AND ANALYSIS

Study design and settings

A prospective controlled study with individual open-label randomisation was designed to compare two groups of outpatients treated with OAA at risk of developing a drug-related iatrogenic event and benefiting from follow-up consultations by an oncologist or haematologist from the Lyon Sud Hospital of the *Hospices Civils de Lyon*, France. Patients randomised to the intervention group will benefit from the ONCORAL programme, with enhanced multidisciplinary management involving hospital and community health professionals while patients randomised to the control group will be managed according to usual care.

For peer review only

Each eligible participant will be recruited following the consultation for an initiation or change of OAA with a hospital oncologist or haematologist. If the patient is ambulatory, deemed to be at important risk of developing drug-related iatrogenic events with OAA, and meets all the inclusion criteria (Table 1), he will be informed orally and with a detailed written notice by the investigator. Randomisation will be set up after obtaining informed consent, in accordance with the principals of good clinical practice.

The allocation of patients to the two study groups will be done via a centralised online randomisation system, in an unbalanced ratio 2:1 between the ONCORAL programme and usual care. Allocation will not be blinded to participants, medical staff, nor the clinical trial staff.

During the course of the study, in the event of a change in treatment and/or complete discontinuation of OAA, the patient will be withdrawn from the study; however, its data will be considered and analysed.

Intervention

Usual care

The patient is usually followed-up by the hospital oncologist or haematologist every month for the first 3 months after OAA initiation and then, if well tolerated, at 3-month intervals. The aim is to ensure the clinical and biological response to the treatment and to monitor tolerance. Changes in prescriptions can occur if needed (e.g. decrease in OAA dosage). There is no intervention from the hospital pharmacist, as OAA is provided by the community pharmacist every month. When dispensing the drug, the community pharmacist may give some advice on how to take it, particularly in relation to other treatments taken by the patient.

The ONCORAL programme

The ONCORAL multidisciplinary programme has been described elsewhere.[20 21] Monthly interviews are carried out with a hospital pharmacist and/or a nurse after each consultation

For peer review only

with the oncologist or haematologist in the hospital or by telephone if no consultation is scheduled.

Briefly, the therapeutic education sessions are personalised, taking into account the specific needs of each patient. They cover issues relating to understanding the medication plan, including all drugs prescribed to the patient, and the management of side effects and the prevention of drug-drug interaction, including self-medication. If the pharmacist detects an interaction, the prescribing physician is contacted to manage it jointly.

All the community health professionals involved in the patient's care (i.e. the attending general practitioner, the community pharmacist, and the home nurse, if any) are informed of the patient's participation in the ONCORAL programme and of all relevant information gathered during the sessions, and are encouraged to share all advice given to the patient using an individual liaison booklet.

Outcome measures and data collection

The main objective of this medico-economic study is to assess the cost-effectiveness, in terms of cost per quality adjusted life years (QALY), of the ONCORAL programme for the drug management monitoring of outpatients treated with OAA and at risk of developing drug-related iatrogenic events compared to usual care.

Primary outcome

The primary outcome will be the estimation of the incremental cost-effectiveness ratio (ICER) at 12 months between the ONCORAL programme and usual care.

The impact of OAA management monitoring will be measured using QALYs that takes into account both survival and quality of life, which is particularly relevant in cancer. The number of QALYs will be assessed using survival time and the 3-level version of the EuroQol 5 Dimensions questionnaire (EQ-5D-3L).[22]

Cost calculations will include hospital-related and ambulatory costs over the 12-month period of the study, from a French health insurance perspective and a hospital perspective.

For peer review only

Secondary outcomes

1. Evaluation of OAA management consequences:
   a. Relative-dose intensity (RDI), defined as the ratio of the prescribed dose of the OAA to the dose recommended in the Summary of Product Characteristics approved as part of the marketing authorisation, with a description of the types and reasons for change in dosage;
   b. Adherence to OAA treatment, measured using a 6-item scale combined with the prescription refill rate;
   c. Number and type of adverse drug events related to OAA and their grade (2, 3, or 4) according to the Common Terminology Criteria for Adverse events (CTCAE v5.0) developed by the US National Cancer Institute;
   d. Number and type of drug-drug interactions related to OAA;
   e. Number and type of proven medication errors, with description of their clinical consequences, according to the National Coordinating Council for Medication Error Reporting and Prevention (NCC MERP).
2. Evaluation of clinical outcomes:
   a. Overall survival and progression-free survival;
   b. Cancer-related quality of life, measured using the EORTC QLQ-C30 questionnaire.
3. Description of patient-reported outcomes (PROs) in relation to their treatment:
   a. Patient satisfaction with OAA treatment, measured by the Treatment Satisfaction with Medicines questionnaire (SATMED-Q);[23]
   b. Patient cognitive and emotional representations of illness, measured by the Brief Illness Perception Questionnaire (Brief IPQ);[24]
   c. Patient cognitive representations of medication, measured by the Beliefs about Medicines Questionnaire (BMQ);[25]
   d. Social support perceived by the patient;[26]

For peer review only

e. Patient health locus of control according to the Therapeutic Self-Care (TSC) measure;[27]

4. Budget impact analysis estimating the annual financial impact of the adoption of the ONCORAL programme on the budget of the French national health insurance system.

5. Description of patient and health professional satisfaction with the ONCORAL programme, measured using a visual analogue scale.

Data collection

The course of the study and the details regarding measurement times for relevant data/questionnaires are presented in Table 2. Patients will be managed according to standard practice for the follow-up of their cancer: 7 visits will be considered for data collection over the 12-month study period, corresponding to the usual consultations with the oncologist or haematologist at the hospital. At each visit, data will be collected from the hospital clinical records, from a dedicated individual booklet filled in by the patient over time, and from the questionnaires. They will be recorded in an electronic case report form. The sponsor will plan on-site data monitoring by clinical research associates, and additional data quality control will be performed throughout the study.

The EQ-5D-3L will be self-administered at baseline and at each follow-up visit. This validated instrument contains 5 dimensions: mobility, self-care, usual activities, pain/discomfort, and anxiety/depression. Each dimension has 3 levels: no problems, some problems, and extreme problems.

The following resource use will be collected for costs estimation: medical consultations and laboratory and imaging examinations, performed on an outpatient basis and during hospital stay/consultations; nursing care at home; time off work; drugs taken on an outpatient basis; emergency room admissions; and hospital admissions related to OAA complications. The hospital information system of the participating centre will be used to complete hospitalisation

For peer review only

data, as well as the French National Health Service data warehouse (ERASME) for ambulatory data.

A specific micro-costing study will be carried out to estimate costs related to the implementation of the ONCORAL programme. Hence, the real time spent by the hospital staff involved will be counted, according to the professional category (oncologist, haematologist, pharmacist, nurse), and will relate to the preparation, realisation, and synthesis for each session; the number of actions carried out towards the community health professionals; and the different additional contacts that could take place with the patient.

Statistical considerations

Sample size calculation

The sample size was based on RDI, as it is associated with the effectiveness of OAA. According to the information available in the literature at the time of the study design, its value was 0.70 in usual care.[28] We assume that the ONCORAL programme will achieve the RDI of 0.85. For an unbalanced 2:1 ratio in favour of the ONCORAL programme, a sample of 130 patients in the ONCORAL group and 65 in the usual care group will achieve a power of 80% at a 0.05 two-sided significance to detect a difference of 0.15 with a standard deviation of 0.35. To take into account early withdrawal from the study, a total of 215 patients will be included in the study (143 in the ONCORAL group and 72 in the usual care group).

The number of patients treated with OAA in the study centre is approximately 350 per year, which will allow the recruitment of the expected number of subjects.

Calculation of costs

The calculation of costs will be performed over a 12-month time horizon, which does not require discounting.

From the hospital perspective, the calculation will be based on production costs (i.e. gross wages plus employer’s costs and social fees) for the time spent by the staff involved in the

For peer review only

ONCORAL programme, cost accounting data at hospital level (consultations, exams, and emergency admissions), and the French national cost studies for hospital stays.

From the French health insurance perspective, the costing will be based on tariffs, i.e. the reimbursement rates for ambulatory resources and diagnosis related groups to estimate the cost for hospital stays. The time spent by the community pharmacists to manage patients included in the ONCORAL programme will be estimated on a sample basis and valued according to expert opinion.

Main analyses

The ICER will be expressed as the extra cost in euros per QALY gained following the introduction of the ONCORAL programme compared to the control group:[29]

$$ICER = \frac{C_{Oncoral} - C_{Control}}{QALY_{Oncoral} - QALY_{Control}}$$

where $C_{Oncoral}$ and $C_{Control}$ are the mean cost per patient in each group, and $QALY_{Oncoral}$ and $QALY_{Control}$ the mean number of QALY in each group.

The principle of calculating QALYs is to weight the time spent in health states by the preference scores associated with these states. Multi-attribute health state classification systems are one of the methods of valuing health states. The EQ-5D-3L self-administered questionnaire is a multi-attribute health state classification system whose preference scores are validated in France.[30] Once the health states are described by the EQ-5D-3L, each of them is individually associated with the corresponding preference score.

A univariate sensitivity analysis will be performed to test the robustness of the cost-effectiveness analysis results and to identify the most sensitive parameters, using a Tornado diagram. The bootstrap method will be used to analyse the sampling uncertainty: 95% confidence intervals of the parameters will be computed; and the joint uncertainty of costs and QALYs will be analysed using cost-effectiveness acceptability curves, which will represent the probability that the ONCORAL programme will be cost-effective at different willingness-to-pay thresholds.

For peer review only

Concerning the primary outcome, a complete case analysis, based on patients for whom all cost and effectiveness data are available, will be performed; then an intention-to-treat analysis will be performed in the whole population after imputation of missing data.

Data regarding secondary outcomes will be described for each group. A Kaplan-Meier model will be used to estimate the overall and the progression-free survival rates up to 12 months in each group, and a log-rank test will be implemented to compare them. Mixed models for repeated measures will be used to estimate the evolution of the different PROs and of cancer-related quality of life.

A pre-established statistical analysis plan will be provided prior to freezing the database and prior to conducting the analyses. All persons involved in data-management, statistical and medico-economic analysis, as well as the principal investigator and the methodologist will have access to the final dataset.

ETHICS AND DISSEMINATION

The study is sponsored by the *Hospices Civils de Lyon*. The protocol has been approved by the Ethics Committee on 2 October 2019 (*Comité de Protection des Personnes Ile-de-France VI;* number 59-19), and by the French national data protection agency (*Commission nationale de l'informatique et des libertés*) in March 2020. Its drafting followed the international recommendations including the SPIRIT guidelines.[31] It was registered on clinicaltrials.gov (NCT03660670). All amendments to the protocol will be submitted for approval and communicated (the current protocol version is version 3 of 26/03/2021). Due to the pandemic episode related to Covid-19, inclusions in the study started in December 2020. To date, 155 patients have been included and randomised. The completion date of the study is expected by December 2024.

All eligible patients are informed and given a written notice explaining the objectives of the study and the terms of their participation; they must consent to their participation before being included. Data management complies with the European general data protection regulation. Data are pseudonymised using a unique identification number for each

For peer review only

participant; only authorised individuals can access the patients' health information. As no risks related to patient participation in the study are expected, there will be no need to set up an independent monitoring committee.

A scientific committee has been set up to validate the final version of the protocol, to supervise the implementation and conduct of the study, and to draft the resulting reports and publications. Trial results will be disseminated at clinical conferences and published in peer-reviewed journals; the guidelines of the International Committee of Medical Journal Editors (ICMJE) will be followed.

For peer review only

DISCUSSION

To our knowledge, this medico-economic study is the first randomised controlled trial to evaluate the cost-effectiveness, using QALYs as an effectiveness outcome measure, of a multidisciplinary medication management programme for outpatients treated with OAA.

A recent review retrieved from the literature that most of the OAA adherence programmes were pharmacy or clinic-based, and dedicated to the initiation and/or implementation phase of OAA therapy.[14] The ONCORAL programme is based on the components known to influence adherence to OAA, such as education, counselling, monitoring of adverse events and drug-drug interactions, adherence follow-up, and a dedicated staff member to be contacted.[32] All sessions are scheduled following a medical consultation with the hospital oncologist, so that patients do not have to return specifically to the hospital, thus reducing the burden. Its originality lies in the involvement of a multidisciplinary hospital team (oncologists, haematologists, pharmacists, and nurses) and the relationship established between them and the community health professionals involved in the follow-up of patients.

Studies reporting pharmacist-led collaborative medication management programmes for OAA have described outcomes to support their benefit, such as patient safety in terms of iatrogenic events, notably drug-related problems that can be identified and solved, and improvement in adherence.[17 33-35] The present study will assess consequences of OAA management in terms of clinical outcomes: the RDI reflects the changes in prescription that

are made as a result of chemotherapy-induced iatrogenic events, and which therefore have an impact on therapeutic effectiveness.[36] The ONCORAL programme should ensure a higher RDI compared to usual care by maintaining adherence to the treatment, notably by educating patients on how to anticipate and manage adverse events. QALYs, which are the remaining years of life for patients and are weighted by a quality of life score, are a particularly important outcome when dealing with cancer patients. QALYs will be used to estimate the cost-effectiveness ratio of the ONCORAL programme compared to usual care, in accordance with the methodological standards of medico-economic studies.[37] To complete the cost analysis of the ONCORAL programme, a micro-costing study will be carried out, providing additional and relevant information for the hospital decision-makers in order to maintain funding.

Another strength of the study is its pragmatic approach, as the ONCORAL programme is already implemented in the participating centre. There will be no additional intervention for the control group that could impact usual care; patients will only be asked to complete the booklet and questionnaires. Despite the deployment of the ONCORAL programme, not all eligible patients can benefit from it today on a routine basis for financial and logistical reasons. A 2:1 ratio was adopted to randomise patients, as this approximates the number of patients currently being monitored by ONCORAL in current practice.

Some limitations can be outlined. First, blinding was not possible, due to the nature of the intervention. It was also not possible to have a blinded primary outcome assessment as its measure is patient reported; QALYs however is known to be a robust measure. Second, individual rather than period randomisation could be a methodological limitation. However, we observed that the probability that 2 patients have the same reference community pharmacy is very low, and patients in the control group are not followed-up at the hospital pharmacy. There is therefore no risk of contamination between groups. Finally, the estimation of the number of patients could not be based on the cost-effectiveness outcome, as the assumption on costs and QALYs could not be found in the literature nor from previous data. We therefore made the estimate on the basis of the most relevant clinical outcome, and

For peer review only

we believe that this will lead to a sufficient number of patients being included to ensure validation of the ICER analysis.

In conclusion, this study will provide new information on the cost-effectiveness of a specific OAA management programme, ONCORAL. In terms of public health, the results of this study could be used as a model and to share experience, especially as it is a simple and transposable programme. Such an evaluation should also help to promote the securing of OAA management by structuring the city-hospital link, which is all the more important as the obstacles to the development of this type of programme are now well-documented, such as the cumbersome nature of implementation, the human resources required, and the lack of funding.[38]

For peer review only

REFERENCES

1. Kantarjian H, O'Brien S, Jabbour E, Garcia-Manero G, Quintas-Cardama A, Shan J, et al. Improved survival in chronic myeloid leukemia since the introduction of imatinib therapy: a single-institution historical experience. Blood 2012;119(9):1981-7.

2. Eek D, Krohe M, Mazar I, Horsfield A, Pompilus F, Friebe R, et al. Patient-reported preferences for oral versus intravenous administration for the treatment of cancer: a review of the literature. Patient Prefer Adherence 2016;10:1609-21.

3. Mc Laughlin AM, Schmulenson E, Teplytska O, Zimmermann S, Opitz P, Groenland SL, et al. Developing a Nationwide Infrastructure for Therapeutic Drug Monitoring of Targeted Oral Anticancer Drugs: The ON-TARGET Study Protocol. Cancers (Basel) 2021;13(24).

4. O'Neill VJ, Twelves CJ. Oral cancer treatment: developments in chemotherapy and beyond. Br J Cancer 2002;87(9):933-7.

5. Jonhson T. Long-Term Care: Safe Drug Handling of Oral Chemotherapy. Consult Pharm 2017;32(2):74-83.

6. Escudero-Vilaplana V, Collado-Borrell R, Hoyo-Munoz A, Gimenez-Manzorro A, Calles A, Osorio S, et al. Potential drug interactions between targeted oral antineoplastic agents and concomitant medication in clinical practice. Expert Opin Drug Saf 2020;19(8):1041-1048.

7. Ranchon F, Bouret C, Charpiat B, Leboucher G. Sécurisation de l'emploi des chimiothérapies anticancéreuses administrables par voie orale. Pharm Hosp Clin 2009;44:36-44.

8. Verbrugghe M, Duprez V, Beeckman D, Grypdonck M, Quaghebeur M, Verschueren C, et al. Factors Influencing Adherence in Cancer Patients Taking Oral Tyrosine Kinase Inhibitors: A Qualitative Study. Cancer Nurs 2016;39(2):153-62.

9. Liu G, Franssen E, Fitch MI, Warner E. Patient preferences for oral versus intravenous palliative chemotherapy. J Clin Oncol 1997;15(1):110-5.

10. Pfeiffer P, Mortensen JP, Bjerregaard B, Eckhoff L, Schonnemann K, Sandberg E, et al. Patient preference for oral or intravenous chemotherapy: a randomised cross-over trial

For peer review only

comparing capecitabine and Nordic fluorouracil/leucovorin in patients with colorectal cancer. Eur J Cancer 2006;42(16):2738-43.

11. Di Nitto M, Sollazzo F, Biagioli V, Pucciarelli G, Torino F, Alvaro R, et al. Self-care behaviors in patients with cancer treated with oral anticancer agents: a systematic review. Support Care Cancer 2022;30(10):8465-8483.
12. Liu S, Kurzrock R. Toxicity of targeted therapy: Implications for response and impact of genetic polymorphisms. Cancer Treat Rev 2014;40(7):883-91.
13. Liu S, Kurzrock R. Understanding Toxicities of Targeted Agents: Implications for Anti-tumor Activity and Management. Semin Oncol 2015;42(6):863-75.
14. Sivakumaran K, Ginex PK, Waseem H, Belcher SM, Lagler-Clark S, LeFebvre KB, et al. Domains of Structured Oral Anticancer Medication Programs: A Scoping Review. Oncol Nurs Forum 2022;49(4):296-306.
15. Canadeo A, Fournogerakis M, Zook F. A Multi-disciplinary Approach to Managing Chronic Myelogenous Leukemia Patients on Oral Anticancer Therapy at a Large Academic Medical Center. Curr Hematol Malig Rep 2021;16(6):509-516.
16. Mathes T, Antoine SL, Pieper D, Eikermann M. Adherence enhancing interventions for oral anticancer agents: a systematic review. Cancer Treat Rev 2014;40(1):102-8.
17. Passey DG, Healy R, Qualls J, Halwani A, Sauer BC. Pharmacist-led collaborative medication management programs for oral antineoplastic therapies: A systematic literature review. J Am Pharm Assoc (2003) 2021;61(3):e7-e18.
18. Zerbit J, Kroemer M, Fuchs B, Detroit M, Decroocq J, Vignon M, et al. Pharmaceutical cancer care for haematology patients on oral anticancer drugs: Findings from an economic, clinical and organisational analysis. Eur J Cancer Care (Engl) 2022;31(6):e13753.
19. Elston Lafata J, Nguyen B, Staresinic C, Johnson M, Gratie D, Muluneh B. Interpersonal communication-, education- and counselling-based interventions to support adherence to oral anticancer therapy: a systematic review. J Oncol Pharm Pract 2022:10781552211073576.

For peer review only

20. Prely H, Herledan C, Caffin AG, Baudouin A, Larbre V, Maire M, et al. Real-life drug-drug and herb-drug interactions in outpatients taking oral anticancer drugs: comparison with databases. J Cancer Res Clin Oncol 2021;148(3):707-718.

21. Collomb B, Dubromel A, Caffin AG, Herledan C, Larbre V, Baudouin A, et al. Assessment of Patient Reported Outcomes (PROs) in Outpatients Taking Oral Anticancer Drugs Included in the Real-Life Oncoral Program. Cancers (Basel) 2022;14(3).

22. Chevalier J, de Pouvourville G. Valuing EQ-5D using time trade-off in France. Eur J Health Econ 2014;14(1):57-66.

23. Ruiz MA, Pardo A, Rejas J, Soto J, Villasante F, Aranguren JL. Development and validation of the "Treatment Satisfaction with Medicines Questionnaire" (SATMED-Q). Value Health 2008;11(5):913-26.

24. Broadbent E, Petrie KJ, Main J, Weinman J. The brief illness perception questionnaire. J Psychosom Res 2006;60(6):631-7.

25. Horne R, Weinman J, Hankins M. The beliefs about medicines questionnaire: The development and evaluation of a new method for assessing the cognitive representation of medication. Psychology & Health 1999;14(1):1-24.

26. Della Vecchia C, Girodet M, Ginguene S, Carpentier C, Leroy T, Simeone A, et al. At the heart of the COVID-19 crisis: Perceived concerns of changes in long-term cancer care in French women with cancer. Eur J Cancer Care (Engl) 2022;31(4):e13599.

27. Sidani S, Doran DI. Development and Validation of a Self-Care Ability Measure. Can J Nurs Res 2014;46(1):11-25.

28. Shirotake S, Yasumizu Y, Ito K, Masunaga A, Ito Y, Miyazaki Y, et al. Impact of Second-Line Targeted Therapy Dose Intensity on Patients With Metastatic Renal Cell Carcinoma. Clin Genitourin Cancer 2016;14(6):e575-e583.

29. Weinstein MC, Torrance G, McGuire A. QALYs: the basics. Value Health 2009;12 Suppl 1:S5-9.

30. Andrade LF, Ludwig K, Goni JMR, Oppe M, de Pouvourville G. A French Value Set for the EQ-5D-5L. Pharmacoeconomics 2020;38(4):413-425.

For peer review only

31. Chan AW, Tetzlaff JM, Gotzsche PC, Altman DG, Mann H, Berlin JA, et al. SPIRIT 2013 explanation and elaboration: guidance for protocols of clinical trials. BMJ 2013;346:e7586.

32. Rosenberg SM, Petrie KJ, Stanton AL, Ngo L, Finnerty E, Partridge AH. Interventions to Enhance Adherence to Oral Antineoplastic Agents: A Scoping Review. J Natl Cancer Inst 2020;112(5):443-465.

33. Ribed A, Romero-Jimenez RM, Escudero-Vilaplana V, Iglesias-Peinado I, Herranz-Alonso A, Codina C, et al. Pharmaceutical care program for onco-hematologic outpatients: safety, efficiency and patient satisfaction. Int J Clin Pharm 2016;38(2):280-8.

34. Clairet AL, Boiteux-Jurain M, Curtit E, Jeannin M, Gerard B, Nerich V, et al. Interaction between phytotherapy and oral anticancer agents: prospective study and literature review. Med Oncol 2019;36(5):45.

35. Durr P, Schlichtig K, Kelz C, Deutsch B, Maas R, Eckart MJ, et al. The Randomized AMBORA Trial: Impact of Pharmacological/Pharmaceutical Care on Medication Safety and Patient-Reported Outcomes During Treatment With New Oral Anticancer Agents. J Clin Oncol 2021;39(18):1983-1994.

36. Hryniuk WM, Bush H. The importance of dose-intensity in chemotherapy of metastatic breast cancer. J Clin Oncol 1984;2:1281-7.

37. Drummond M, Weatherly H, Ferguson B. Economic evaluation of health interventions. BMJ 2008;337:a1204.

38. Occhipinti S, Petit-Jean E, Pinguet F, Beaupin C, Daouphars M, Parent D, et al. Pharmacist involvement in supporting care in patients receiving oral anticancer therapies: A situation report in French cancer centers. Bull Cancer 2017;104(9):727-734.

For peer review only

ACKNOWLEDGEMENTS

We thank Véréna Landel, from the *Direction de la Recherche en Santé* of the *Hospices Civils de Lyon* for her help in manuscript preparation.

AUTHORS' CONTRIBUTIONS

LH, PG, GD and CR designed the study; LH, PG and GD proposed the methodology and the medico-economic component; AGC, CH and FR participated in the development of the study.

LH, PG, and CR drafted the manuscript; GD, AGC, CH and FR participated in drafting of the manuscript. All authors have read and approved the final manuscript.

FUNDING

The French Ministry of Health (DGOS) supported this work (PRME-17-0487). The funding body had no role in the design of the study.

COMPETING INTERESTS

The authors declare that they have no competing interests.

PATIENT AND PUBLIC INVOLVEMENT

Patients and/or public were not involved in the development of the research question or the design of the study.

For peer review only

Table 1: Patient eligibility criteria

Inclusion criteria	Exclusion criteria
- Adult over 18 years of age	- Treatment with OAA under a clinical trial or compassionate use
- Diagnosed cancer	- Treatment managed at home by a caregiver exclusively
- Ambulatory status (not hospitalised for the management and treatment of the cancer)	- No declared attending physician
- Initiation or change of OAA (indication in the context of the marketing authorisation)	- No or ≥ 2 declared regular community pharmacies
- Sufficient autonomy to manage the treatment at home*	- Participation in a clinical trial that may modify the costs of care
- No major psychiatric cognitive disorder likely to interfere with the ONCORAL programme*	- Legal protection or institutionalisation
- Considered by the oncologist to be at risk of drug-related adverse events, or to have 3 or more of the following risk factors for developing a drug-related adverse event:<br>• ≥ 2 OAA prescribed in combination<br>• ≥ 2 lines of treatment<br>• Combination with an injectable chemotherapy protocol<br>• Discontinuous OAA regimen<br>• ≥ 2 associated chronic conditions<br>• ≥ 5 associated drugs including OAA<br>• Creatinine clearance < 60 ml/min<br>• Frailties and psychosocial conditions (isolated patient, foreigner, limited autonomy)	

* According to the treating oncologist
OAA = oral anticancer agent

For peer review only

Table 2: Overview of the course of the study

Study period	*Enrolment & Allocation*	*Allocation*													*Close out*
Timepoint	D_0	D_8 † +/- 3d	D_{15} +/- 3d	D_{22} +/- 3d	M_1 +/- 8d	M_{1+15i} † +/- 3d	M_2 +/- 8d	M_{2+15d} † +/- 3d	M_3 +/- 8d	M_4†, M_5† +/- 8d	M_6 +/- 15d	M_7, M_8 +/- 15d	M_9 +/- 15d	M_{10}, M_{11} +/- 15d	M_{12} +/- 15d
On-site visit	V1				V_2		V_3		V_4		V_5		V_6		V_7
**ENROLMENT:**															
Informed consent	X														
Randomisation	X														
Background and socio-demographic information	X														
Patient booklet (delivery, then collection of the data)	X				X		X		X		X		X		X
**INTERVENTION: ONCORAL educational session***	X	X			X	X	X	X	X	X	X		X		X
Information on treatment by the community pharmacist in conjunction with the hospital pharmacist*	X				X		X		X		X		X		X
Information on treatment by the community pharmacist*										X		X		X	
Micro-costing*	X	X	X	X	X	X	X	X	X	X	X	X	X	X	X
**ASSESSMENTS:**															
Resource consumption					X		X		X		X		X		X
Prescription changes for the oral anticancer drug, if any					X		X		X		X		X		X
Adherence scale									X		X				X
Prescription refill rate									X		X				X
Adverse events		X*	X*	X*	X	X*	X	X*	X		X		X		X
Medication errors					X		X		X		X		X		X
Drug-drug interactions					X*		X*		X*		X*		X*		X
EQ-5D-3L	X				X		X		X		X		X		X
EORTC QLQ-C30	X								X		X				X
SatMed Q					X				X		X		X		X
Brief-IPQ	X										X				X
BMQ	X										X				X
Perceived social support	X										X				X

Health locus of control questionnaire	X										X				X
Vital status					X		X		X		X		X		X
Patient and health professional satisfaction with the ONCORAL programme*															X

* Only for patients randomised in the intervention group (ONCORAL programme)
† Patients in the intervention group are reached by phone for this session
BMQ = Beliefs about Medicines Questionnaire. Brief IPQ = Brief Illness Perception Questionnaire. EQ-5D-3L = EuroQool-5Dimension questionnaire. OAA = Oral anticancer agents. SATMED-Q = Treatment Satisfaction with Medicines questionnaire. V = Visit.

For peer review only

Reporting checklist for protocol of a clinical trial.

Based on the SPIRIT guidelines.

Instructions to authors

Complete this checklist by entering the page numbers from your manuscript where readers will find each of the items listed below.

Your article may not currently address all the items on the checklist. Please modify your text to include the missing information. If you are certain that an item does not apply, please write "n/a" and provide a short explanation.

Upload your completed checklist as an extra file when you submit to a journal.

In your methods section, say that you used the SPIRITreporting guidelines, and cite them as:

Chan A-W, Tetzlaff JM, Gøtzsche PC, Altman DG, Mann H, Berlin J, Dickersin K, Hróbjartsson A, Schulz KF, Parulekar WR, Krleža-Jerić K, Laupacis A, Moher D. SPIRIT 2013 Explanation and Elaboration: Guidance for protocols of clinical trials. BMJ. 2013;346:e7586

		Reporting Item	Page Number
**Administrative information**			
Title	#1	Descriptive title identifying the study design, population, interventions, and, if applicable, trial acronym	1

For peer review only

Trial registration	#2a	Trial identifier and registry name. If not yet registered, name of intended registry	3; 13
Trial registration: data set	#2b	All items from the World Health Organization Trial Registration Data Set	n/a
Protocol version	#3	Date and version identifier	13
Funding	#4	Sources and types of financial, material, and other support	21
Roles and responsibilities: contributorship	#5a	Names, affiliations, and roles of protocol contributors	1; 21
Roles and responsibilities: sponsor contact information	#5b	Name and contact information for the trial sponsor	13
Roles and responsibilities: sponsor and funder	#5c	Role of study sponsor and funders, if any, in study design; collection, management, analysis, and interpretation of data; writing of the report; and the decision to submit the report for publication, including whether they will have ultimate authority over any of these activities	21
Roles and responsibilities: committees	#5d	Composition, roles, and responsibilities of the coordinating centre, steering committee, endpoint adjudication committee, data management team, and	14

For peer review only

		other individuals or groups overseeing the trial, if applicable (see Item 21a for data monitoring committee)	
**Introduction**			
Background and rationale	#6a	Description of research question and justification for undertaking the trial, including summary of relevant studies (published and unpublished) examining benefits and harms for each intervention	5-6
Background and rationale: choice of comparators	#6b	Explanation for choice of comparators	5-6
Objectives	#7	Specific objectives or hypotheses	6
Trial design	#8	Description of trial design including type of trial (eg, parallel group, crossover, factorial, single group), allocation ratio, and framework (eg, superiority, equivalence, non-inferiority, exploratory)	6
**Methods: Participants, interventions, and outcomes**			
Study setting	#9	Description of study settings (eg, community clinic, academic hospital) and list of countries where data will be collected. Reference to where list of study sites can be obtained	6-7

For peer review only

Eligibility criteria	#10	Inclusion and exclusion criteria for participants. If applicable, eligibility criteria for study centres and individuals who will perform the interventions (eg, surgeons, psychotherapists)	6; 22 (Table 1)
Interventions: description	#11a	Interventions for each group with sufficient detail to allow replication, including how and when they will be administered	7-8
Interventions: modifications	#11b	Criteria for discontinuing or modifying allocated interventions for a given trial participant (eg, drug dose change in response to harms, participant request, or improving / worsening disease)	7
Interventions: adherance	#11c	Strategies to improve adherence to intervention protocols, and any procedures for monitoring adherence (eg, drug tablet return; laboratory tests)	n/a
Interventions: concomitant care	#11d	Relevant concomitant care and interventions that are permitted or prohibited during the trial	7-8
Outcomes	#12	Primary, secondary, and other outcomes, including the specific measurement variable (eg, systolic blood pressure), analysis metric (eg, change from baseline, final value, time to event), method of aggregation (eg, median, proportion), and time point for each outcome. Explanation of the clinical relevance of chosen efficacy and harm outcomes is strongly recommended	8-10

For peer review only

Participant timeline	#13	Time schedule of enrolment, interventions (including any run-ins and washouts), assessments, and visits for participants. A schematic diagram is highly recommended (see Figure)	23-24 (Table 2)
Sample size	#14	Estimated number of participants needed to achieve study objectives and how it was determined, including clinical and statistical assumptions supporting any sample size calculations	11
Recruitment	#15	Strategies for achieving adequate participant enrolment to reach target sample size	11
**Methods: Assignment of interventions (for controlled trials)**			
Allocation: sequence generation	#16a	Method of generating the allocation sequence (eg, computer-generated random numbers), and list of any factors for stratification. To reduce predictability of a random sequence, details of any planned restriction (eg, blocking) should be provided in a separate document that is unavailable to those who enrol participants or assign interventions	7
Allocation concealment mechanism	#16b	Mechanism of implementing the allocation sequence (eg, central telephone; sequentially numbered, opaque,	7

For peer review only

		sealed envelopes), describing any steps to conceal the sequence until interventions are assigned	
Allocation: implementation	#16c	Who will generate the allocation sequence, who will enrol participants, and who will assign participants to interventions	7
Blinding (masking)	#17a	Who will be blinded after assignment to interventions (eg, trial participants, care providers, outcome assessors, data analysts), and how	n/a
Blinding (masking): emergency unblinding	#17b	If blinded, circumstances under which unblinding is permissible, and procedure for revealing a participant's allocated intervention during the trial	n/a
**Methods: Data collection, management, and analysis**			
Data collection plan	#18a	Plans for assessment and collection of outcome, baseline, and other trial data, including any related processes to promote data quality (eg, duplicate measurements, training of assessors) and a description of study instruments (eg, questionnaires, laboratory tests) along with their reliability and validity, if known. Reference to where data collection forms can be found, if not in the protocol	10-11

For peer review only

Data collection plan: retention	#18b	Plans to promote participant retention and complete follow-up, including list of any outcome data to be collected for participants who discontinue or deviate from intervention protocols	n/a
Data management	#19	Plans for data entry, coding, security, and storage, including any related processes to promote data quality (eg, double data entry; range checks for data values). Reference to where details of data management procedures can be found, if not in the protocol	10
Statistics: outcomes	#20a	Statistical methods for analysing primary and secondary outcomes. Reference to where other details of the statistical analysis plan can be found, if not in the protocol	11-13
Statistics: additional analyses	#20b	Methods for any additional analyses (eg, subgroup and adjusted analyses)	n/a
Statistics: analysis population and missing data	#20c	Definition of analysis population relating to protocol non-adherence (eg, as randomised analysis), and any statistical methods to handle missing data (eg, multiple imputation)	13

Methods: Monitoring

Data monitoring: formal committee	#21a	Composition of data monitoring committee (DMC); summary of its role and reporting structure; statement of whether it is independent from the sponsor and competing interests; and reference to where further	13

For peer review only

		details about its charter can be found, if not in the protocol. Alternatively, an explanation of why a DMC is not needed	
Data monitoring: interim analysis	#21b	Description of any interim analyses and stopping guidelines, including who will have access to these interim results and make the final decision to terminate the trial	n/a
Harms	#22	Plans for collecting, assessing, reporting, and managing solicited and spontaneously reported adverse events and other unintended effects of trial interventions or trial conduct	n/a
Auditing	#23	Frequency and procedures for auditing trial conduct, if any, and whether the process will be independent from investigators and the sponsor	10
**Ethics and dissemination**			
Research ethics approval	#24	Plans for seeking research ethics committee / institutional review board (REC / IRB) approval	13
Protocol amendments	#25	Plans for communicating important protocol modifications (eg, changes to eligibility criteria, outcomes, analyses) to relevant parties (eg, investigators, REC / IRBs, trial participants, trial registries, journals, regulators)	13

For peer review only

Consent or assent	#26a	Who will obtain informed consent or assent from potential trial participants or authorised surrogates, and how (see Item 32)	7;13
Consent or assent: ancillary studies	#26b	Additional consent provisions for collection and use of participant data and biological specimens in ancillary studies, if applicable	n/a
Confidentiality	#27	How personal information about potential and enrolled participants will be collected, shared, and maintained in order to protect confidentiality before, during, and after the trial	13
Declaration of interests	#28	Financial and other competing interests for principal investigators for the overall trial and each study site	21
Data access	#29	Statement of who will have access to the final trial dataset, and disclosure of contractual agreements that limit such access for investigators	13
Ancillary and post trial care	#30	Provisions, if any, for ancillary and post-trial care, and for compensation to those who suffer harm from trial participation	n/a
Dissemination policy: trial results	#31a	Plans for investigators and sponsor to communicate trial results to participants, healthcare professionals, the public, and other relevant groups (eg, via publication, reporting in results databases, or other data sharing arrangements), including any publication restrictions	14

For peer review only

Dissemination policy: authorship	#31b	Authorship eligibility guidelines and any intended use of professional writers	14
Dissemination policy: reproducible research	#31c	Plans, if any, for granting public access to the full protocol, participant-level dataset, and statistical code	n/a

Appendices

Informed consent materials	#32	Model consent form and other related documentation given to participants and authorised surrogates	n/a
Biological specimens	#33	Plans for collection, laboratory evaluation, and storage of biological specimens for genetic or molecular analysis in the current trial and for future use in ancillary studies, if applicable	n/a

None The SPIRIT Explanation and Elaboration paper is distributed under the terms of the Creative Commons Attribution License CC-BY-NC. This checklist can be completed online using https://www.goodreports.org/, a tool made by the EQUATOR Network in collaboration with Penelope.ai

For peer review only

BMJ Open

Cost-effectiveness of the ONCORAL multidisciplinary programme for the management of outpatients taking oral anticancer agents at risk of drug related event: protocol for a pragmatic randomised controlled study

Journal:	*BMJ Open*
Manuscript ID	bmjopen-2023-074956.R1
Article Type:	Protocol
Date Submitted by the Author:	11-Dec-2023
Complete List of Authors:	Huot, Laure; Hospices Civils de Lyon, Pole Sante Publique; Université Lyon 1, U1290 INSERM RESHAPE Research on Healthcare Performance<br>Guerre, Pascale; Hospices Civils de Lyon, Pôle Santé Publique, Service Evaluation Economique en Santé; Université Lyon 1, UR4129 Parcours Systémique en Santé P2S<br>DESCOTES, Guillaume; Hospices Civils de Lyon, Unité de Pharmacie Clinique Oncologique<br>CAFFIN, Anne-Gaëlle; Hospices Civils de Lyon, Unité de Pharmacie Clinique Oncologique<br>HERLEDAN, Chloé; Hospices Civils de Lyon, Unité de Pharmacie Clinique Oncologique; Université Lyon 1, EA3738 CICLY Centre pour l'innovation et la cancérologie de Lyon1<br>Ranchon, Florence; Hospices Civils de Lyon, Groupement Hospitalier Sud, Clinical Oncology Pharmacy Department; Université Lyon 1, EA3738 CICLY Centre pour l'innovation et la cancérologie de Lyon1<br>Rioufol, Catherine; Hospices Civils de Lyon, Groupement Hospitalier Sud, Clinical Oncology Pharmacy Department; Université Lyon 1, EMR 3738
**Primary Subject Heading**:	Oncology
Secondary Subject Heading:	Health economics
Keywords:	Adult oncology < ONCOLOGY, Health economics < HEALTH SERVICES ADMINISTRATION & MANAGEMENT, Quality of Life

SCHOLARONE™
Manuscripts

TITLE: Cost-effectiveness of the ONCORAL multidisciplinary programme for the management of outpatients taking oral anticancer agents at risk of drug related event: protocol for a pragmatic randomised controlled study

AUTHORS: Laure HUOT[1,2], Pascale GUERRE[1,3], Guillaume DESCOTES[4], Anne-Gaëlle CAFFIN[4], Chloé HERLEDAN[4.5], Florence RANCHON[4.5,†], Catherine RIOUFOL[4.5,†]

1. Hospices Civils de Lyon, Pôle de Santé Publique, Service Evaluation Economique en Santé, F-69003 Lyon, France

2. Université Lyon 1, Inserm U1290 Research on Healthcare Performance (RESHAPE), F-69008 Lyon, France

3. Université Lyon 1, Laboratoire Parcours Systémique en Santé, F-69003 Lyon, France

4. Hospices Civils de Lyon, Unité de Pharmacie Clinique Oncologique, F-69495 Pierre-Bénite, France

5. Université Lyon 1, CICLY Centre pour l'Innovation et la Cancérologie de Lyon 1-EA3738, F-69921 Lyon, France

† Both authors contributed equally

Corresponding author:

Pr. Catherine RIOUFOL

Hospices Civils de Lyon, Hôpital Lyon Sud, Service pharmaceutique, Unité de Pharmacie Clinique Oncologique, 165 Chemin du Grand Revoyet, F-69495 PIERRE-BENITE, FRANCE

catherine.rioufol@chu-lyon.fr

KEYWORDS

Oral anticancer agent (OAA); Medication management programme; Study protocol; Cost-effectiveness

For peer review only

WORD COUNT: 3 598

For peer review only

ABSTRACT

Introduction

The development of oral anticancer agents (OAA) has profoundly changed cancer care, leading patients to manage their chemotherapy treatment on an outpatient basis. The prevention of iatrogenic effects of OAA remains a major concern, especially since their side effects are not less serious than those of intravenous chemotherapy. The ONCORAL programme was set up to secure the management of OAA in cancer patients followed at the Lyon University Hospital. This multidisciplinary programme involves hospital pharmacists, nurses, oncologists, and haematologists, as well as community health professionals. Given the economic stakes that this programme entails for the health system, a medico-economic study was designed.

Methods and analysis

This is a prospective controlled study, with individual open-label randomisation. A total of 216 outpatients treated with OAA and at risk of developing a drug-related iatrogenic event, will be randomised (2:1) to undergo follow-up in the ONCORAL programme or usual care. The primary outcome will be the estimation of the incremental cost-effectiveness ratio (difference in total costs per quality adjusted life years gained) at 12 months between the 2 groups. The secondary outcomes will be evaluation of OAA management consequences (relative-dose intensity, adherence, adverse drug events, drug-drug interactions, and proven medication errors), evaluation of overall survival and cancer-related quality of life, and patient reported outcomes in relation to the treatment. A budget impact analysis will be implemented. Patient and health professional satisfaction regarding the ONCORAL programme will be measured.

Ethics and dissemination

Approval to conduct this study was obtained from an Ethics Committee (*Comité de Protection des Personnes Ile-de-France VI*) in October 2019, and from the French data protection agency (*Commission Nationale de l'Informatique et des Libertés*), according to the

For peer review only

French Law. Trial results will be disseminated at clinical conferences and published in peer-reviewed journals.

Trial registration: NCT03660670; recruitment started in December 2020.

For peer review only

STRENGTHS AND LIMITATIONS OF THIS STUDY

- This study is the first cost-effectiveness evaluation of a multidisciplinary medication management programme for adult cancer outpatients treated with OAA.
- A pragmatic open-label randomised design was adopted, based on a programme implemented in a French University Hospital, involving hospital health professionals (oncologists, haematologists, pharmacists, and nurses) and based on a structured relationship with the community health professionals who follow the patient.
- The cost-effectiveness will be estimated at 12 months, by comparing the difference in total costs and quality of life-adjusted years (QALYs) gained between the ONCORAL programme and usual care.
- Due to the nature of the interventions, patients and professionals participating in the study are not blinded to management allocation.

For peer review only

INTRODUCTION

For many years, the treatment of cancer patients remained essentially hospital-based, relying on surgery, radiotherapy, and injectable chemotherapy. Since the early 2000's, oral anticancer agents (OAA), mostly targeted therapies such as tyrosine kinase inhibitors, have been steadily developed, contributing to significantly improve the prognosis of cancer patients.(1)

By allowing patients previously treated by injectable chemotherapy to receive oral therapy, the use of OAA has shifted patient management from hospital and healthcare provider administered treatment to self-administered treatment at home.(2) Despite their high price, OAA were expected to reduce certain healthcare costs and were considered for their convenience and ease of administration.(3, 4)

However, these drugs expose patients to iatrogenic risks, in part due to their narrow therapeutic index and complex pharmacological profile.(5) They may also result in drug-related problems such as medication errors, adverse effects, and drug-drug interactions, which have been reported in half of the patients and considered as major in almost 20%.(6) Importantly, OAA adherence may be reduced due to these drug-related problems and may be further complicated by the administration regimen, such as a discontinuous dosing regimen or combination with another OAA or injectable chemotherapy.(7, 8) In this context, the prevention of iatrogenic effects is crucial to enhance safety. Furthermore, as drug-related problems lead to an over-consumption of healthcare resources, this could lead to an increase in patient management-related costs.

It has been reported that patients show a preference for OAA over injectable chemotherapy, as the former meets their expectations in terms of comfort and autonomy.(9, 10) Their perception of efficacy and past treatments experience (including toxicity) may also influence this preference.(2) It has been shown that for patients to successfully manage their OAA treatment, self-care support and multidisciplinary education across healthcare settings are

For peer review only

needed.(11) Finally, such medication management contributes to the optimal effectiveness of the treatment and a better quality of life.(12, 13)

Structured OAA programmes have been set up in different countries and described in the literature, based on different components and organisation,(14) highlighting the follow-up and monitoring of patients throughout their treatment.(15) A promising effect has been described in the literature for certain adherence enhancing interventions, applied to target one or multiple factors influencing patient behaviour.(16) The management of side effects should be more specifically included in OAA care programmes in order to help patients better identify them and improve their self-care behaviour.(11) Although studies have also assessed the economic impact of pharmacist-led medication management programmes, showing cost savings and cost avoidance,(17, 18) none has evaluated the efficiency of such interventions on the whole patient pathway. There is thus still a need for rigorous studies to assess educational and counselling practices supporting adherence to OAA in terms of clinical benefit and efficiency.(19)

We set up a medico-economic study to evaluate, with a high level of evidence, the cost-effectiveness of a multidisciplinary OAA management programme for outpatients with cancer followed in a French university hospital.

METHODS AND ANALYSIS

Study design and settings

A prospective controlled study with individual open-label randomisation was designed to compare two groups of outpatients treated with OAA at risk of developing a drug-related iatrogenic event and benefiting from follow-up consultations by an oncologist or haematologist from the Lyon Sud Hospital of the *Hospices Civils de Lyon*, France. Patients randomised to the intervention group will benefit from the ONCORAL programme, with enhanced multidisciplinary management involving hospital and community health

For peer review only

professionals while patients randomised to the control group will be managed according to usual care.

Each eligible participant will be recruited following the consultation for an initiation or change of OAA with a hospital oncologist or haematologist. If the patient is ambulatory, deemed to be at important risk of developing drug-related iatrogenic events with OAA, and meets all the inclusion criteria (Table 1), he will be informed orally and with a detailed written notice by the investigator. Randomisation will be set up after obtaining informed consent, in accordance with the principals of good clinical practice.

The allocation of patients to the two study groups will be done via a centralised online randomisation system, in an unbalanced ratio 2:1 between the ONCORAL programme and usual care. Allocation will not be blinded to participants, medical staff, nor the clinical trial staff.

During the course of the study, in the event of a change in treatment and/or complete discontinuation of OAA, the patient will be withdrawn from the study; however, its data will be considered and analysed.

Intervention

Usual care

The patient is usually followed-up by the hospital oncologist or haematologist every month for the first 3 months after OAA initiation and then, if well tolerated, at 3-month intervals. The aim is to ensure the clinical and biological response to the treatment and to monitor tolerance. Changes in prescriptions can occur if needed (e.g. decrease in OAA dosage). There is no intervention from the hospital pharmacist, as OAA is provided by the community pharmacist every month. When dispensing the drug, the community pharmacist may give some advice on how to take it, particularly in relation to other treatments taken by the patient.

The ONCORAL programme

For peer review only

The ONCORAL multidisciplinary programme has been described elsewhere.(20, 21) Monthly interviews are carried out with a hospital pharmacist and/or a nurse after each consultation with the oncologist or haematologist in the hospital or by telephone if no consultation is scheduled. It is carried out for the entire duration of the OAA treatment and will be assessed in the context of the study up to 12 months after beginning of treatment (i.e. patient's inclusion).

Briefly, the therapeutic education sessions are personalised, taking into account the specific needs of each patient. They cover issues relating to understanding the medication plan, including all drugs prescribed to the patient, and the management of side effects and the prevention of drug-drug interaction, including self-medication. If the pharmacist detects an interaction, the prescribing physician is contacted to manage it jointly.

All the community health professionals involved in the patient's care (i.e. the attending general practitioner, the community pharmacist, and the home nurse, if any) are informed of the patient's participation in the ONCORAL programme and of all relevant information gathered during the sessions, and are encouraged to share all advice given to the patient using an individual liaison booklet.

Outcome measures and data collection

The main objective of this medico-economic study is to assess the cost-effectiveness, in terms of cost per quality adjusted life years (QALY), of the ONCORAL programme for the drug management monitoring of outpatients treated with OAA and at risk of developing drug-related iatrogenic events compared to usual care.

Primary outcome

The primary outcome will be the estimation of the incremental cost-effectiveness ratio (ICER) at 12 months between the ONCORAL programme and usual care.

For peer review only

The impact of OAA management monitoring will be measured using QALYs that takes into account both survival and quality of life, which is particularly relevant in cancer. The number of QALYs will be assessed using survival time and the 3-level version of the EuroQol 5 Dimensions questionnaire (EQ-5D-3L).(22)

Cost calculations will include hospital-related and ambulatory costs over the 12-month period of the study, from a French health insurance perspective and a hospital perspective.

Secondary outcomes

1. Evaluation of OAA management consequences:
    a. Relative-dose intensity (RDI), defined as the ratio of the prescribed dose of the OAA to the dose recommended in the Summary of Product Characteristics approved as part of the marketing authorisation, with a description of the types and reasons for change in dosage;
    b. Adherence to OAA treatment, measured using a 6-item scale combined with the prescription refill rate;
    c. Number and type of adverse drug events related to OAA and their grade (2, 3, or 4) according to the Common Terminology Criteria for Adverse events (CTCAE v5.0) developed by the US National Cancer Institute;
    d. Number and type of drug-drug interactions related to OAA;
    e. Number and type of proven medication errors, with description of their clinical consequences, according to the National Coordinating Council for Medication Error Reporting and Prevention (NCC MERP).
2. Evaluation of clinical outcomes:
    a. Overall survival and progression-free survival;
    b. Cancer-related quality of life, measured using the EORTC QLQ-C30 questionnaire.
3. Description of patient-reported outcomes (PROs) in relation to their treatment:

For peer review only

a. Patient satisfaction with OAA treatment, measured by the Treatment Satisfaction with Medicines questionnaire (SATMED-Q);(23)
b. Patient cognitive and emotional representations of illness, measured by the Brief Illness Perception Questionnaire (Brief IPQ);(24)
c. Patient cognitive representations of medication, measured by the Beliefs about Medicines Questionnaire (BMQ);(25)
d. Social support perceived by the patient;(26)
e. Patient health locus of control according to the Therapeutic Self-Care (TSC) measure;(27)

4. Budget impact analysis estimating the annual financial impact of the adoption of the ONCORAL programme on the budget of the French national health insurance system.
5. Description of patient and health professional satisfaction with the ONCORAL programme, measured using a visual analogue scale.

Data collection

The course of the study and the details regarding measurement times for relevant data/questionnaires are presented in online supplemental table. Patients will be managed according to standard practice for the follow-up of their cancer: 7 visits will be considered for data collection over the 12-month study period, corresponding to the usual consultations with the oncologist or haematologist at the hospital. At each visit, data will be collected from the hospital clinical records, from a dedicated individual booklet filled in by the patient over time, and from the questionnaires. They will be recorded in an electronic case report form. On-site data monitoring will be planned, and additional data quality control will be performed throughout the study.

The EQ-5D-3L will be self-administered at baseline and at each follow-up visit. This validated instrument contains 5 dimensions: mobility, self-care, usual activities, pain/discomfort, and

For peer review only

anxiety/depression. Each dimension has 3 levels: no problems, some problems, and extreme problems.

The following resource use will be collected for costs estimation: medical consultations and laboratory and imaging examinations, performed on an outpatient basis and during hospital stay/consultations; nursing care at home; time off work; drugs taken on an outpatient basis; emergency room admissions; and hospital admissions related to OAA complications. The hospital information system of the participating centre will be used to complete hospitalisation data, as well as the French National Health Service data warehouse (ERASME) for ambulatory data.

A specific micro-costing study will be carried out to estimate costs related to the implementation of the ONCORAL programme. Hence, the real time spent by the additional hospital staff required for the intervention will be counted, according to the professional category (i.e. pharmacist, nurse). This will be self-reported and will relate to the preparation, realisation, and synthesis for each session; the number of actions carried out towards the community health professionals; and the different additional contacts that could take place with the patient.

Statistical considerations

Sample size calculation

The sample size was based on RDI, as it is associated with the effectiveness of OAA. According to the information available in the literature at the time of the study design, its value was 0.70 in usual care.(28) We assume that the ONCORAL programme will achieve the RDI of 0.85. For an unbalanced 2:1 ratio in favour of the ONCORAL programme, a sample of 130 patients in the ONCORAL group and 65 in the usual care group will achieve a power of 80% at a 0.05 two-sided significance to detect a difference of 0.15 with a standard deviation of 0.35. To take into account early withdrawal from the study, a total of 215 patients will be included in the study (143 in the ONCORAL group and 72 in the usual care group).

For peer review only

The number of patients treated with OAA in the study centre is approximately 350 per year, which will allow the recruitment of the expected number of subjects.

Calculation of costs

The calculation of costs will be performed over a 12-month time horizon, which does not require discounting.

From the hospital perspective, the calculation will be based on production costs (i.e. gross wages plus employer's costs and social fees) for the time spent by the staff involved in the ONCORAL programme, cost accounting data at hospital level (consultations, exams, and emergency admissions), and the French national cost studies for hospital stays.

From the French health insurance perspective, the costing will be based on tariffs, i.e. the reimbursement rates for ambulatory resources and diagnosis related groups to estimate the cost for hospital stays. The time spent by the community pharmacists to manage patients included in the ONCORAL programme will be estimated on a sample basis and valued according to expert opinion.

Main analyses

The ICER will be expressed as the extra cost in euros per QALY gained following the introduction of the ONCORAL programme compared to the control group:(29)

$$ICER = \frac{C_{Oncoral} - C_{Control}}{QALY_{Oncoral} - QALY_{Control}}$$

where $C_{Oncoral}$ and $C_{Control}$ are the mean cost per patient in each group, and $QALY_{Oncoral}$ and $QALY_{Control}$ the mean number of QALY in each group.

The principle of calculating QALYs is to weight the time spent in health states by the preference scores associated with these states. Multi-attribute health state classification systems are one of the methods of valuing health states. The EQ-5D-3L self-administered questionnaire is a multi-attribute health state classification system whose preference scores

For peer review only

are validated in France.(30) Once the health states are described by the EQ-5D-3L, each of them is individually associated with the corresponding preference score.

A univariate sensitivity analysis will be performed to test the robustness of the cost-effectiveness analysis results and to identify the most sensitive parameters, using a Tornado diagram. The bootstrap method will be used to analyse the sampling uncertainty: 95% confidence intervals of the parameters will be computed; and the joint uncertainty of costs and QALYs will be analysed using cost-effectiveness acceptability curves, which will represent the probability that the ONCORAL programme will be cost-effective at different willingness-to-pay thresholds.

Concerning the primary outcome, a complete case analysis, based on patients for whom all cost and effectiveness data are available, will be performed; an intention-to-treat analysis will be performed in the whole population after imputation of missing data.

Data regarding secondary outcomes will be described for each group. Missing data will be documented but there will be no imputation for secondary outcomes. A Kaplan-Meier model will be used to estimate the overall and the progression-free survival rates up to 12 months in each group, and a log-rank test will be implemented to compare them. Mixed models for repeated measures will be used to estimate the evolution of the different PROs and of cancer-related quality of life.

A pre-established statistical analysis plan will be provided prior to freezing the database and prior to conducting the analyses. All persons involved in data-management, statistical and medico-economic analysis, as well as the principal investigator and the methodologist will have access to the final dataset.

ETHICS AND DISSEMINATION

The study is funded by a public grant from the French Ministry of Health and sponsored by the *Hospices Civils de Lyon*, a teaching hospital, as responsible for its management. The protocol has been approved by the Ethics Committee on 2 October 2019 (*Comité de*

For peer review only

Protection des Personnes Ile-de-France VI; number 59-19), and by the French national data protection agency (*Commission nationale de l'informatique et des libertés*) in March 2020. Its drafting followed the international recommendations including the SPIRIT guidelines.(31) It was registered on clinicaltrials.gov (NCT03660670). All amendments to the protocol will be submitted for approval and communicated (the current protocol version is version 3 of 26/03/2021). Due to the pandemic episode related to Covid-19, inclusions in the study started in December 2020. To date, 184 patients have been included and randomised. The completion date of the study is expected by December 2024.

All eligible patients are informed and given a written notice explaining the objectives of the study and the terms of their participation; they must consent to their participation before being included. Data management complies with the European general data protection regulation. Data are pseudonymised using a unique identification number for each participant; only authorised individuals can access the patients' health information. As no risks related to patient participation in the study are expected, there will be no need to set up an independent monitoring committee.

A scientific committee has been set up to validate the final version of the protocol, to supervise the implementation and conduct of the study, and to draft the resulting reports and publications. Trial results will be disseminated at clinical conferences and published in peer-reviewed journals; the guidelines of the International Committee of Medical Journal Editors (ICMJE) will be followed.

DISCUSSION

To our knowledge, this medico-economic study is the first randomised controlled trial to evaluate the cost-effectiveness, using QALYs as an effectiveness outcome measure, of a multidisciplinary medication management programme for outpatients treated with OAA.

A recent review retrieved from the literature that most of the OAA adherence programmes were pharmacy or clinic-based, and dedicated to the initiation and/or implementation phase

For peer review only

of OAA therapy.(14) The ONCORAL programme is based on the components known to influence adherence to OAA, such as education, counselling, monitoring of adverse events and drug-drug interactions, adherence follow-up, and a dedicated staff member to be contacted.(32) All sessions are scheduled following a medical consultation with the hospital oncologist, so that patients do not have to return specifically to the hospital, thus reducing the burden. Its originality lies in the involvement of a multidisciplinary hospital team (oncologists, haematologists, pharmacists, and nurses) and the relationship established between them and the community health professionals involved in the follow-up of patients.

Studies reporting pharmacist-led collaborative medication management programmes for OAA have described outcomes to support their benefit, such as patient safety in terms of iatrogenic events, notably drug-related problems that can be identified and solved, and improvement in adherence.(17, 33-35) The present study will assess consequences of OAA management in terms of clinical outcomes: the RDI reflects the changes in prescription that are made as a result of chemotherapy-induced iatrogenic events, and which therefore have an impact on therapeutic effectiveness.(36) The ONCORAL programme should ensure a higher RDI compared to usual care by maintaining adherence to the treatment, notably by educating patients on how to anticipate and manage adverse events. QALYs, which are the remaining years of life for patients and are weighted by a quality of life score, are a particularly important outcome when dealing with cancer patients. QALYs will be used to estimate the cost-effectiveness ratio of the ONCORAL programme compared to usual care, in accordance with the methodological standards of medico-economic studies.(37) To complete the cost analysis of the ONCORAL programme, a micro-costing study will be carried out from the hospital perspective, since it is the hospital that is currently financing the additional human resources required for this intervention. This analysis will provide additional and relevant information for hospital decision-makers in order to maintain funding, but also for the French insurance system if payment of a dedicated sum could be considered to extend this intervention to the national level.

For peer review only

Another strength of the study is its pragmatic approach, as the ONCORAL programme is already implemented in the participating centre. There will be no additional intervention for the control group that could impact usual care; patients will only be asked to complete the booklet and questionnaires. Despite the deployment of the ONCORAL programme, not all eligible patients can benefit from it today on a routine basis for financial and logistical reasons. A 2:1 ratio was adopted to randomise patients, as this approximates the number of patients currently being monitored by ONCORAL in current practice.

At the time the study was designed, and to the best of our knowledge, the ONCORAL programme was the only one in France to combine multidisciplinary intervention aimed at all cancer patients with OAA, with a link to community healthcare professionals. Despite the monocentric design of the study, we believe that, if the results are in line with our hypothesis, the ONCORAL programme could be disseminated to centres wishing to implement this intervention. A first step in this direction has already been taken with the introduction of a national experimental care pathway to support French patients taking oral therapies at home.

Some limitations can be outlined. First, blinding was not possible, due to the nature of the intervention. It was also not possible to have a blinded primary outcome assessment as its measure is patient reported; QALYs however is known to be a robust measure. Second, individual rather than period randomisation could be a methodological limitation. However, we observed that the probability that 2 patients have the same reference community pharmacy is very low, and patients in the control group are not followed-up at the hospital pharmacy. There is therefore no risk of contamination between groups. Finally, the estimation of the number of patients could not be based on the cost-effectiveness outcome, as the assumption on costs and QALYs could not be found in the literature nor from previous data. We therefore made the estimate on the basis of the most relevant clinical outcome, and we believe that this will lead to a sufficient number of patients being included to ensure validation of the ICER analysis.

For peer review only

In conclusion, this study will provide new information on the cost-effectiveness of a specific OAA management programme, ONCORAL. In terms of public health, the results of this study could be used as a model and to share experience, especially as it is a simple and transposable programme. Such an evaluation should also help to promote the securing of OAA management by structuring the city-hospital link, which is all the more important as the obstacles to the development of this type of programme are now well-documented, such as the cumbersome nature of implementation, the human resources required, and the lack of funding.(38)

For peer review only

REFERENCES

1. Kantarjian H, O'Brien S, Jabbour E, Garcia-Manero G, Quintas-Cardama A, Shan J, et al. Improved survival in chronic myeloid leukemia since the introduction of imatinib therapy: a single-institution historical experience. Blood 2012;119(9):1981-7.

2. Eek D, Krohe M, Mazar I, Horsfield A, Pompilus F, Friebe R, et al. Patient-reported preferences for oral versus intravenous administration for the treatment of cancer: a review of the literature. Patient Prefer Adherence 2016;10:1609-21.

3. Mc Laughlin AM, Schmulenson E, Teplytska O, Zimmermann S, Opitz P, Groenland SL, et al. Developing a Nationwide Infrastructure for Therapeutic Drug Monitoring of Targeted Oral Anticancer Drugs: The ON-TARGET Study Protocol. Cancers (Basel) 2021;13(24).

4. O'Neill VJ, Twelves CJ. Oral cancer treatment: developments in chemotherapy and beyond. Br J Cancer 2002;87(9):933-7.

5. Jonhson T. Long-Term Care: Safe Drug Handling of Oral Chemotherapy. Consult Pharm 2017;32(2):74-83.

6. Escudero-Vilaplana V, Collado-Borrell R, Hoyo-Munoz A, Gimenez-Manzorro A, Calles A, Osorio S, et al. Potential drug interactions between targeted oral antineoplastic agents and concomitant medication in clinical practice. Expert Opin Drug Saf 2020;19(8):1041-1048.

7. Ranchon F, Bouret C, Charpiat B, Leboucher G. Sécurisation de l'emploi des chimiothérapies anticancéreuses administrables par voie orale. Pharm Hosp Clin 2009;44:36-44.

8. Verbrugghe M, Duprez V, Beeckman D, Grypdonck M, Quaghebeur M, Verschueren C, et al. Factors Influencing Adherence in Cancer Patients Taking Oral Tyrosine Kinase Inhibitors: A Qualitative Study. Cancer Nurs 2016;39(2):153-62.

9. Liu G, Franssen E, Fitch MI, Warner E. Patient preferences for oral versus intravenous palliative chemotherapy. J Clin Oncol 1997;15(1):110-5.

For peer review only

10. Pfeiffer P, Mortensen JP, Bjerregaard B, Eckhoff L, Schonnemann K, Sandberg E, et al. Patient preference for oral or intravenous chemotherapy: a randomised cross-over trial comparing capecitabine and Nordic fluorouracil/leucovorin in patients with colorectal cancer. Eur J Cancer 2006;42(16):2738-43.

11. Di Nitto M, Sollazzo F, Biagioli V, Pucciarelli G, Torino F, Alvaro R, et al. Self-care behaviors in patients with cancer treated with oral anticancer agents: a systematic review. Support Care Cancer 2022;30(10):8465-8483.

12. Liu S, Kurzrock R. Toxicity of targeted therapy: Implications for response and impact of genetic polymorphisms. Cancer Treat Rev 2014;40(7):883-91.

13. Liu S, Kurzrock R. Understanding Toxicities of Targeted Agents: Implications for Anti-tumor Activity and Management. Semin Oncol 2015;42(6):863-75.

14. Sivakumaran K, Ginex PK, Waseem H, Belcher SM, Lagler-Clark S, LeFebvre KB, et al. Domains of Structured Oral Anticancer Medication Programs: A Scoping Review. Oncol Nurs Forum 2022;49(4):296-306.

15. Canadeo A, Fournogerakis M, Zook F. A Multi-disciplinary Approach to Managing Chronic Myelogenous Leukemia Patients on Oral Anticancer Therapy at a Large Academic Medical Center. Curr Hematol Malig Rep 2021;16(6):509-516.

16. Mathes T, Antoine SL, Pieper D, Eikermann M. Adherence enhancing interventions for oral anticancer agents: a systematic review. Cancer Treat Rev 2014;40(1):102-8.

17. Passey DG, Healy R, Qualls J, Halwani A, Sauer BC. Pharmacist-led collaborative medication management programs for oral antineoplastic therapies: A systematic literature review. J Am Pharm Assoc (2003) 2021;61(3):e7-e18.

18. Zerbit J, Kroemer M, Fuchs B, Detroit M, Decroocq J, Vignon M, et al. Pharmaceutical cancer care for haematology patients on oral anticancer drugs: Findings from an economic, clinical and organisational analysis. Eur J Cancer Care (Engl) 2022;31(6):e13753.

19. Elston Lafata J, Nguyen B, Staresinic C, Johnson M, Gratie D, Muluneh B. Interpersonal communication-, education- and counselling-based interventions to support adherence to

For peer review only

oral anticancer therapy: a systematic review. J Oncol Pharm Pract 2022:10781552211073576.

20. Prely H, Herledan C, Caffin AG, Baudouin A, Larbre V, Maire M, et al. Real-life drug-drug and herb-drug interactions in outpatients taking oral anticancer drugs: comparison with databases. J Cancer Res Clin Oncol 2021;148(3):707-718.

21. Collomb B, Dubromel A, Caffin AG, Herledan C, Larbre V, Baudouin A, et al. Assessment of Patient Reported Outcomes (PROs) in Outpatients Taking Oral Anticancer Drugs Included in the Real-Life Oncoral Program. Cancers (Basel) 2022;14(3).

22. Chevalier J, de Pouvourville G. Valuing EQ-5D using time trade-off in France. Eur J Health Econ 2014;14(1):57-66.

23. Ruiz MA, Pardo A, Rejas J, Soto J, Villasante F, Aranguren JL. Development and validation of the "Treatment Satisfaction with Medicines Questionnaire" (SATMED-Q). Value Health 2008;11(5):913-26.

24. Broadbent E, Petrie KJ, Main J, Weinman J. The brief illness perception questionnaire. J Psychosom Res 2006;60(6):631-7.

25. Horne R, Weinman J, Hankins M. The beliefs about medicines questionnaire: The development and evaluation of a new method for assessing the cognitive representation of medication. Psychology & Health 1999;14(1):1-24.

26. Della Vecchia C, Girodet M, Ginguene S, Carpentier C, Leroy T, Simeone A, et al. At the heart of the COVID-19 crisis: Perceived concerns of changes in long-term cancer care in French women with cancer. Eur J Cancer Care (Engl) 2022;31(4):e13599.

27. Sidani S, Doran DI. Development and Validation of a Self-Care Ability Measure. Can J Nurs Res 2014;46(1):11-25.

28. Shirotake S, Yasumizu Y, Ito K, Masunaga A, Ito Y, Miyazaki Y, et al. Impact of Second-Line Targeted Therapy Dose Intensity on Patients With Metastatic Renal Cell Carcinoma. Clin Genitourin Cancer 2016;14(6):e575-e583.

29. Weinstein MC, Torrance G, McGuire A. QALYs: the basics. Value Health 2009;12 Suppl 1:S5-9.

For peer review only

30. Andrade LF, Ludwig K, Goni JMR, Oppe M, de Pouvourville G. A French Value Set for the EQ-5D-5L. Pharmacoeconomics 2020;38(4):413-425.

31. Chan AW, Tetzlaff JM, Gotzsche PC, Altman DG, Mann H, Berlin JA, et al. SPIRIT 2013 explanation and elaboration: guidance for protocols of clinical trials. BMJ 2013;346:e7586.

32. Rosenberg SM, Petrie KJ, Stanton AL, Ngo L, Finnerty E, Partridge AH. Interventions to Enhance Adherence to Oral Antineoplastic Agents: A Scoping Review. J Natl Cancer Inst 2020;112(5):443-465.

33. Ribed A, Romero-Jimenez RM, Escudero-Vilaplana V, Iglesias-Peinado I, Herranz-Alonso A, Codina C, et al. Pharmaceutical care program for onco-hematologic outpatients: safety, efficiency and patient satisfaction. Int J Clin Pharm 2016;38(2):280-8.

34. Clairet AL, Boiteux-Jurain M, Curtit E, Jeannin M, Gerard B, Nerich V, et al. Interaction between phytotherapy and oral anticancer agents: prospective study and literature review. Med Oncol 2019;36(5):45.

35. Durr P, Schlichtig K, Kelz C, Deutsch B, Maas R, Eckart MJ, et al. The Randomized AMBORA Trial: Impact of Pharmacological/Pharmaceutical Care on Medication Safety and Patient-Reported Outcomes During Treatment With New Oral Anticancer Agents. J Clin Oncol 2021;39(18):1983-1994.

36. Hryniuk WM, Bush H. The importance of dose-intensity in chemotherapy of metastatic breast cancer. J Clin Oncol 1984;2:1281-7.

37. Drummond M, Weatherly H, Ferguson B. Economic evaluation of health interventions. BMJ 2008;337:a1204.

38. Occhipinti S, Petit-Jean E, Pinguet F, Beaupin C, Daouphars M, Parent D, et al. Pharmacist involvement in supporting care in patients receiving oral anticancer therapies: A situation report in French cancer centers. Bull Cancer 2017;104(9):727-734.

For peer review only

ACKNOWLEDGEMENTS

We thank Véréna Landel, from the *Direction de la Recherche en Santé* of the *Hospices Civils de Lyon* for her help in manuscript preparation.

AUTHORS' CONTRIBUTIONS

LH, PG, GD and CR designed the study; LH, PG and GD proposed the methodology and the medico-economic component; AGC, CH and FR participated in the development of the study.

LH, PG, and CR drafted the manuscript; GD, AGC, CH and FR participated in drafting of the manuscript. All authors have read and approved the final manuscript.

FUNDING

The French Ministry of Health (DGOS) supported this work (PRME-17-0487). The funding body had no role in the design of the study.

COMPETING INTERESTS

The authors declare that they have no competing interests.

PATIENT AND PUBLIC INVOLVEMENT

Patients and/or public were not involved in the development of the research question or the design of the study.

For peer review only

Table 1: Patient eligibility criteria

Inclusion criteria	Exclusion criteria
- Adult over 18 years of age	- Treatment with OAA under a clinical trial or compassionate use
- Diagnosed cancer	- Treatment managed at home by a caregiver exclusively
- Ambulatory status (not hospitalised for the management and treatment of the cancer)	- No declared attending physician
- Initiation or change of OAA (indication in the context of the marketing authorisation)	- No or ≥ 2 declared regular community pharmacies
- Sufficient autonomy to manage the treatment at home*	- Participation in a clinical trial that may modify the costs of care
- No major psychiatric cognitive disorder likely to interfere with the ONCORAL programme*	- Legal protection or institutionalisation
- Considered by the oncologist to be at risk of drug-related adverse events, or to have 3 or more of the following risk factors for developing a drug-related adverse event:<br>• ≥ 2 OAA prescribed in combination<br>• ≥ 2 lines of treatment<br>• Combination with an injectable chemotherapy protocol<br>• Discontinuous OAA regimen<br>• ≥ 2 associated chronic conditions<br>• ≥ 5 associated drugs including OAA<br>• Creatinine clearance < 60 ml/min<br>• Frailties and psychosocial conditions (isolated patient, foreigner, limited autonomy)	

* According to the treating oncologist
OAA = oral anticancer agent

For peer review only

Supplementary table: Overview of the course of the study

STUDY PERIOD	*Enrolment & Allocation*	*Allocation*													*Close out*
TIMEPOINT	D_0	D_8 † +/- 3d	D_{15} +/- 3d	D_{22} +/- 3d	M_1 +/- 8d	M_{1+15j} † +/- 3d	M_2 +/- 8d	M_{2+15d} † +/- 3d	M_3 +/- 8d	M_4†, M_5† +/- 8d	M_6 +/- 15d	M_7, M_8 +/- 15d	M_9 +/- 15d	M_{10}, M_{11} +/- 15d	M_{12} +/- 15d
ON-SITE VISIT	*V1*				V_2		V_3		V_4		V_5		V_6		V_7
**ENROLMENT:**															
Informed consent	X														
Randomisation	X														
Background and socio-demographic information	X														
Patient booklet (delivery, then collection of the data)	X				X		X		X		X		X		X
**INTERVENTION: ONCORAL educational session***	X	X			X	X	X	X	X	X	X		X		X
Information on treatment by the community pharmacist in conjunction with the hospital pharmacist*	X				X		X		X		X		X		X
Information on treatment by the community pharmacist*										X		X		X	
Micro-costing*	X	X	X	X	X	X	X	X	X	X	X	X	X	X	X
**ASSESSMENTS:**															
Resource consumption					X		X		X		X		X		X
Prescription changes for the oral anticancer drug, if any					X		X		X		X		X		X
Adherence scale									X		X				X
Prescription refill rate									X		X				X
Adverse events		X*	X*	X*	X	X*	X	X*	X		X		X		X
Medication errors					X		X		X		X		X		X
Drug-drug interactions					X*		X*		X*		X*		X*		X
EQ-5D-3L	X				X		X		X		X		X		X
EORTC QLQ-C30	X								X		X				X
SatMed Q					X				X		X		X		X
Brief-IPQ	X										X				X

BMQ	X										X				X
Perceived social support	X										X				X
Health locus of control questionnaire	X										X				X
Vital status					X		X		X		X		X		X
Patient and health professional satisfaction with the ONCORAL programme*															X

* Only for patients randomised in the intervention group (ONCORAL programme)
† Patients in the intervention group are reached by phone for this session
BMQ = Beliefs about Medicines Questionnaire. Brief IPQ = Brief Illness Perception Questionnaire. EQ-5D-3L = EuroQool-5Dimension questionnaire. OAA = Oral anticancer agents. SATMED-Q = Treatment Satisfaction with Medicines questionnaire. V = Visit.

For peer review only

Reporting checklist for protocol of a clinical trial.

Based on the SPIRIT guidelines.

Instructions to authors

Complete this checklist by entering the page numbers from your manuscript where readers will find each of the items listed below.

Your article may not currently address all the items on the checklist. Please modify your text to include the missing information. If you are certain that an item does not apply, please write "n/a" and provide a short explanation.

Upload your completed checklist as an extra file when you submit to a journal.

In your methods section, say that you used the SPIRITreporting guidelines, and cite them as:

Chan A-W, Tetzlaff JM, Gøtzsche PC, Altman DG, Mann H, Berlin J, Dickersin K, Hróbjartsson A, Schulz KF, Parulekar WR, Krleža-Jerić K, Laupacis A, Moher D. SPIRIT 2013 Explanation and Elaboration: Guidance for protocols of clinical trials. BMJ. 2013;346:e7586

		Reporting Item	Page Number
**Administrative information**			
Title	#1	Descriptive title identifying the study design, population, interventions, and, if applicable, trial acronym	1

For peer review only

Trial registration	#2a	Trial identifier and registry name. If not yet registered, name of intended registry	3; 13
Trial registration: data set	#2b	All items from the World Health Organization Trial Registration Data Set	n/a
Protocol version	#3	Date and version identifier	13
Funding	#4	Sources and types of financial, material, and other support	13; 21
Roles and responsibilities: contributorship	#5a	Names, affiliations, and roles of protocol contributors	1; 21
Roles and responsibilities: sponsor contact information	#5b	Name and contact information for the trial sponsor	13
Roles and responsibilities: sponsor and funder	#5c	Role of study sponsor and funders, if any, in study design; collection, management, analysis, and interpretation of data; writing of the report; and the decision to submit the report for publication, including whether they will have ultimate authority over any of these activities	13; 21
Roles and responsibilities: committees	#5d	Composition, roles, and responsibilities of the coordinating centre, steering committee, endpoint adjudication committee, data management team,	14

For peer review only

		and other individuals or groups overseeing the trial, if applicable (see Item 21a for data monitoring committee)	
**Introduction**			
Background and rationale	#6a	Description of research question and justification for undertaking the trial, including summary of relevant studies (published and unpublished) examining benefits and harms for each intervention	5-6
Background and rationale: choice of comparators	#6b	Explanation for choice of comparators	5-6
Objectives	#7	Specific objectives or hypotheses	6
Trial design	#8	Description of trial design including type of trial (eg, parallel group, crossover, factorial, single group), allocation ratio, and framework (eg, superiority, equivalence, non-inferiority, exploratory)	6
**Methods: Participants, interventions, and outcomes**			
Study setting	#9	Description of study settings (eg, community clinic, academic hospital) and list of countries where data	6-7

For peer review only

		will be collected. Reference to where list of study sites can be obtained	
Eligibility criteria	#10	Inclusion and exclusion criteria for participants. If applicable, eligibility criteria for study centres and individuals who will perform the interventions (eg, surgeons, psychotherapists)	6; 22 (Table 1)
Interventions: description	#11a	Interventions for each group with sufficient detail to allow replication, including how and when they will be administered	7-8
Interventions: modifications	#11b	Criteria for discontinuing or modifying allocated interventions for a given trial participant (eg, drug dose change in response to harms, participant request, or improving / worsening disease)	7
Interventions: adherance	#11c	Strategies to improve adherence to intervention protocols, and any procedures for monitoring adherence (eg, drug tablet return; laboratory tests)	n/a
Interventions: concomitant care	#11d	Relevant concomitant care and interventions that are permitted or prohibited during the trial	7-8
Outcomes	#12	Primary, secondary, and other outcomes, including the specific measurement variable (eg, systolic blood pressure), analysis metric (eg, change from baseline, final value, time to event), method of aggregation (eg, median, proportion), and time point for each outcome. Explanation of the clinical	8-10

For peer review only

		relevance of chosen efficacy and harm outcomes is strongly recommended	
Participant timeline	#13	Time schedule of enrolment, interventions (including any run-ins and washouts), assessments, and visits for participants. A schematic diagram is highly recommended (see Figure)	11; Supplementary table
Sample size	#14	Estimated number of participants needed to achieve study objectives and how it was determined, including clinical and statistical assumptions supporting any sample size calculations	11
Recruitment	#15	Strategies for achieving adequate participant enrolment to reach target sample size	11
**Methods: Assignment of interventions (for controlled trials)**			
Allocation: sequence generation	#16a	Method of generating the allocation sequence (eg, computer-generated random numbers), and list of any factors for stratification. To reduce predictability of a random sequence, details of any planned restriction (eg, blocking) should be provided in a separate document that is	7

For peer review only

		unavailable to those who enrol participants or assign interventions	
Allocation concealment mechanism	#16b	Mechanism of implementing the allocation sequence (eg, central telephone; sequentially numbered, opaque, sealed envelopes), describing any steps to conceal the sequence until interventions are assigned	7
Allocation: implementation	#16c	Who will generate the allocation sequence, who will enrol participants, and who will assign participants to interventions	7
Blinding (masking)	#17a	Who will be blinded after assignment to interventions (eg, trial participants, care providers, outcome assessors, data analysts), and how	n/a
Blinding (masking): emergency unblinding	#17b	If blinded, circumstances under which unblinding is permissible, and procedure for revealing a participant's allocated intervention during the trial	n/a
**Methods: Data collection, management, and analysis**			
Data collection plan	#18a	Plans for assessment and collection of outcome, baseline, and other trial data, including any related processes to promote data quality (eg, duplicate measurements, training of assessors) and a	10-11

For peer review only

		description of study instruments (eg, questionnaires, laboratory tests) along with their reliability and validity, if known. Reference to where data collection forms can be found, if not in the protocol	
Data collection plan: retention	#18b	Plans to promote participant retention and complete follow-up, including list of any outcome data to be collected for participants who discontinue or deviate from intervention protocols	n/a
Data management	#19	Plans for data entry, coding, security, and storage, including any related processes to promote data quality (eg, double data entry; range checks for data values). Reference to where details of data management procedures can be found, if not in the protocol	10
Statistics: outcomes	#20a	Statistical methods for analysing primary and secondary outcomes. Reference to where other details of the statistical analysis plan can be found, if not in the protocol	11-13
Statistics: additional analyses	#20b	Methods for any additional analyses (eg, subgroup and adjusted analyses)	n/a
Statistics: analysis population and missing data	#20c	Definition of analysis population relating to protocol non-adherence (eg, as randomised analysis), and	13

For peer review only

		any statistical methods to handle missing data (eg, multiple imputation)	
**Methods: Monitoring**			
Data monitoring: formal committee	#21a	Composition of data monitoring committee (DMC); summary of its role and reporting structure; statement of whether it is independent from the sponsor and competing interests; and reference to where further details about its charter can be found, if not in the protocol. Alternatively, an explanation of why a DMC is not needed	13
Data monitoring: interim analysis	#21b	Description of any interim analyses and stopping guidelines, including who will have access to these interim results and make the final decision to terminate the trial	n/a
Harms	#22	Plans for collecting, assessing, reporting, and managing solicited and spontaneously reported adverse events and other unintended effects of trial interventions or trial conduct	n/a
Auditing	#23	Frequency and procedures for auditing trial conduct, if any, and whether the process will be independent from investigators and the sponsor	10
**Ethics and dissemination**			

For peer review only

Research ethics approval	#24	Plans for seeking research ethics committee / institutional review board (REC / IRB) approval	13
Protocol amendments	#25	Plans for communicating important protocol modifications (eg, changes to eligibility criteria, outcomes, analyses) to relevant parties (eg, investigators, REC / IRBs, trial participants, trial registries, journals, regulators)	13
Consent or assent	#26a	Who will obtain informed consent or assent from potential trial participants or authorised surrogates, and how (see Item 32)	7;13
Consent or assent: ancillary studies	#26b	Additional consent provisions for collection and use of participant data and biological specimens in ancillary studies, if applicable	n/a
Confidentiality	#27	How personal information about potential and enrolled participants will be collected, shared, and maintained in order to protect confidentiality before, during, and after the trial	13
Declaration of interests	#28	Financial and other competing interests for principal investigators for the overall trial and each study site	21
Data access	#29	Statement of who will have access to the final trial dataset, and disclosure of contractual agreements that limit such access for investigators	13

For peer review only

Ancillary and post trial care	#30	Provisions, if any, for ancillary and post-trial care, and for compensation to those who suffer harm from trial participation	n/a
Dissemination policy: trial results	#31a	Plans for investigators and sponsor to communicate trial results to participants, healthcare professionals, the public, and other relevant groups (eg, via publication, reporting in results databases, or other data sharing arrangements), including any publication restrictions	14
Dissemination policy: authorship	#31b	Authorship eligibility guidelines and any intended use of professional writers	14
Dissemination policy: reproducible research	#31c	Plans, if any, for granting public access to the full protocol, participant-level dataset, and statistical code	n/a
**Appendices**			
Informed consent materials	#32	Model consent form and other related documentation given to participants and authorised surrogates	n/a
Biological specimens	#33	Plans for collection, laboratory evaluation, and storage of biological specimens for genetic or molecular analysis in the current trial and for future use in ancillary studies, if applicable	n/a

For peer review only

None The SPIRIT Explanation and Elaboration paper is distributed under the terms of the Creative Commons Attribution License CC-BY-NC. This checklist can be completed online using https://www.goodreports.org/, a tool made by the EQUATOR Network in collaboration with Penelope.ai

For peer review only
